# Chronic Pain and Complementary Therapies: The Challenge of Measuring Patient Benefit

**DOI:** 10.1111/jep.70740

**Published:** 2026-11-12

**Authors:** Carole A. Paley, Matthew R. Mulvey, Mark I. Johnson

**Affiliations:** ^1^ Patient Centred Outcomes Research Group (PCOR), Leeds Institute of Medical Research, Level 5, Clinical Sciences Building St James' University Hospital Leeds UK; ^2^ Centre for Pain Research, School of Health, Portland Way Leeds Beckett University Leeds UK; ^3^ Academic Unit of Palliative Care, Leeds Institute of Health Sciences Leeds University School of Medicine Leeds UK

**Keywords:** chronic pain, complementary therapies, logic model, outcome measures

## Abstract

**Rationale:**

Chronic pain remains a major challenge in healthcare despite advances in pharmaceutical management. Growing recognition of the biopsychosocial complexity of chronic pain has highlighted the need for approaches that reflect this multidimensional experience. Complementary therapies, used alongside mainstream treatments, are increasingly popular, yet evidence supporting their efficacy is limited. Randomised controlled trials (RCTs) have not generated findings sufficient to influence policy or practice, potentially because their outcome measures fail to capture what matters most to patients.

**Methods:**

A keyword search of Ovid Medline, PubMed, the Cochrane Collaboration, and National Institute for Health and Care Excellence (NICE) guidance from 2010 onwards identified contemporary chronic primary pain guidelines and systematic reviews.

**Results:**

Our narrative synthesis examines current approaches to chronic pain management, the prevalence of complementary therapy use, and the evidence underpinning efficacy. We critically consider whether RCT designs and reductionist outcome measures are appropriate for evaluating holistic interventions.

**Conclusions:**

We argue that the unique components of complementary therapies must be understood as potential mediators of change. Shifting focus from narrow efficacy testing towards evaluating meaning, patient responses, and perceived utility may provide more relevant insights. Developing programme theories and logic models can clarify how complementary therapies work and guide the selection of suitable outcome measures for future research.

## 1 Introduction

Chronic pain, defined by the International Association for the Study of Pain (IASP), is pain that persists or recurs for longer than 3 months [1, 2]. It is thought to affect between a third and a half of the UK population [3] and varies globally from 9.9% to 50.3% [4]. Chronic pain has a significant negative impact on wellbeing and quality of life [5] and also has economic repercussions in terms of working days lost and the cost of healthcare interventions [6]. It has been estimated that the cost to the UK economy of lost working days amounts to an estimated £7 billion per year [7]. Musculoskeletal conditions as a whole cost the National Health Service (NHS) approximately £4.76 billion per year and take up an estimated 30% of the time of general practitioners [8, 9].

Over many decades, the focus of the clinical management of chronic primary pain has been on pharmaceutical approaches, including opioids, which helped precipitate the opioid crisis, but has been to the detriment of exploring the efficacy and effectiveness of non‐pharmacological alternatives [10]. The most recent National Institute for Health and Care Excellence (NICE) guidance no longer recommends the use of opioid or non‐steroidal anti‐inflammatory medication for chronic primary pain; instead, non‐pharmacological interventions such as exercise is recommended as a first‐line approach, followed by consideration of antidepressants [11]. For patients who are presumed to have non‐malignant nociceptive pain, healthcare professionals are advised to consider alternative approaches to pain management such as exercise and psychological approaches (e.g., cognitive‐behavioural therapy). Complementary therapies, such as acupuncture, massage and mindful meditation are also used as holistic, non‐pharmaceutical adjuncts to manage pain and other symptoms. These interventions are used alongside mainstream medicine but not used as an alternative to it.

Despite an acknowledgment of the role of complementary therapies in the management of chronic pain, a tension persists between the fundamental human right to pain relief [12], and the need to tackle the problem of opioid overuse [13]. An outdated model of chronic pain remains, predicated on the view that pain can be described simply in terms of neurological mechanisms and therefore treated with agents that modify neuronal activity. This model has prevailed over the past 200 years in western medicine, and yet we still see an increasing incidence of chronic pain, which suggests that the biomedical model does not adequately describe the whole pain experience. It fundamentally lacks any description of the emotional, cognitive, spiritual, social and environmental components, often called the ‘lived experience’ of pain. Complementary therapies, unlike pharmaceutical interventions, are holistic and do address the lived experience of pain and its multiple biopsychosocial components.

Patients with chronic pain are often highly motivated to relieve their pain by any means; tested and untested, conventional and unconventional. Many choose to explore complementary therapies as holistic approaches, to treat and manage pain, even though RCT efficacy evidence is conflicting and inconclusive [14]. There exists a longstanding gap between little or no scientific evidence and numerous (often anecdotal) reports of substantial benefit from both patients and practitioners [15]. Despite the lack of supporting evidence on which to base a judgement of efficacy, complementary therapies remain popular with patients experiencing chronic pain and other long‐term conditions, including poor mental health [16, 17] and some patients and practitioners report significant (if anecdotal) benefit.

The aim of this article is to explore the unique benefits of complementary therapies through the experiences of patients with chronic primary non‐cancer pain, and to discuss how these benefits could be evaluated. Our approach will be to provide a narrative synthesis of recent published literature to determine the relative popularity of complementary therapies in individuals living with chronic pain and give a contemporary perspective on the evaluation of benefit for chronic primary and non‐cancer pain conditions. We will discuss use of the ‘gold‐standard’ RCT design for evaluating efficacy of complementary therapies and whether the outcome measures commonly used are too reductionist to capture their effects on chronic pain which itself is a multifaceted experience.

## 2 Methods

### 2.1 Scope of This Review

This article is a narrative review intended to explore conceptual and methodological issues surrounding the evaluation of complementary therapies in chronic pain. While systematic search methods were used to identify relevant literature, the objective was not to undertake a formal systematic review or meta‐analysis.

Although examples are drawn from various persistent pain conditions, the principal focus of this discussion is chronic primary and chronic non‐cancer pain conditions. Cancer pain has not been included because it introduces different contexts, priorities and goals which might not be present with non‐malignant pain.

Several examples of complementary therapies have been included which fall within the main groupings of manual/touch therapies, mind‐body therapies and activity‐based therapies.

### 2.2 Information Sources

Key word literature searches were conducted using several electronic databases: Ovid MEDLINE, PubMed and The Cochrane Collaboration. Guidance on the treatment of chronic primary pain issued by NICE was also included. Searches were conducted for literature published between 2000 and 2025.

### 2.3 Search Strategy

The search strategy detailed below was designed as informal and evolved as the study progressed

**Table:** 

Element	Strategy Details
Electronic databases	Ovid MEDLINE, PubMed and The Cochrane Collaboration
Other literature	NICE guidelines and other grey literature
Publication dates	2000–2025
Language	English language only
Concept 1 (Condition)	Chronic primary pain, chronic non‐cancer pain, persistent pain, musculoskeletal pain
Concept 2 (Intervention)	Complementary therapies, CAM, TCAM, CAIM, Integrative therapies/medicine
Concept 3 (methodology/outcomes)	Patient‐reported outcome measures, PROMS, pain assessment, methodological challenges, qualitative outcomes
Inclusion criteria	All studies, including clinical trials, mixed methods studies and qualitative methodologies.
Exclusion criteria	Animal studies, studies using alternative medicine, including ingested products or indwelling materials, studies not primarily focusing on complementary therapies.

Selection of studies was conducted by CAP and MRM, and where relevance to the review was uncertain, MIJ acted as arbiter. A narrative review methodology was chosen to facilitate exploration of conceptual and methodological issues surrounding the evaluation of complementary therapies in chronic pain. While systematic search methods were used to identify relevant literature, the objective was not to undertake a formal systematic review or meta‐analysis. The primary aim was to provide a broad but flexible interpretative synthesis of a multifaceted topic by examining diverse methodologies and concepts across heterogeneous forms of literature. Accordingly, synthesis was thematic and exploratory, allowing themes and lines of discussion to emerge iteratively from the literature. This approach informed a novel framework for considering the evaluation of complementary therapies. The methodological quality of included studies was not formally assessed, although issues relating to study quality were discussed where relevant to the narrative.

## 3 Description, Prevalence, and Popularity of Complementary Therapies

### 3.1 Description and Definition

Complementary therapies have been described as interventions which provide an addition to conventional, or mainstream medicine, rather than replacing it [18]. For the purposes of this article, we have defined complementary therapies as separate and distinct from alternative medicine. In the literature, complementary medicine falls under the general umbrella of complementary and alternative medicine (CAM), complementary and integrative medicine (CAIM) or traditional, complementary, and alternative medicine (TCAM), although many of these acronyms are used interchangeably. For the remainder of this article, we are focusing on complementary therapies which are delivered specifically as an adjunct to conventional medicine, but do not involve ingestion of substances (e.g., herbal remedies) or act as a replacement for medical interventions.

Examples of commonly used complementary therapies, using our definition, along with their usual means of delivery are described in Table 1. We have broadly classified them into three categories, although we acknowledge that there is some overlap between them.

**Table 1:** A broad classification of complementary therapies.

Category of intervention	Complementary therapy	Usual approach
Manual techniques/touch therapies	Massage	Therapist‐delivered
	Reflexology	Therapist‐delivered
	Acupuncture	Therapist‐delivered
Mind‐body therapies*	Aromatherapy**	Self, or therapist‐delivered
	Mindfulness/meditation	Self, or therapist‐delivered
	Yoga, Tai Chi or Qi Gong	Self, or therapist‐delivered
Activity‐based*	Creative arts therapies(includes music, art, dance)	Therapist‐delivered(can be continued by participant).

* Some therapies within these categories can also be delivered via audio, video or online approaches and in groups or individually.

** Sometimes combined with massage.

### 3.2 The Popularity of Complementary Therapies

A systematic review of national surveys of the use of traditional, complementary and alternative medicine between 2010 and 2019 revealed a wide range of prevalence (24%–71.3%) for any reason (clinical or otherwise) across 14 countries including the UK, the US, Canada, Australia, Europe, the Middle East, China, and South Korea [19]. It was noted, however, that many studies lacked an operational definition of these therapies and there was a high degree of heterogeneity in prevalence time frames, data collection methods and methods of analysis. It was also unclear where the boundaries lay between complementary approaches and conventional medicine or foodstuffs across studies. In England, a national household survey suggested that 26.3% of the population use complementary therapies, with the most popular therapies being massage, aromatherapy and acupuncture/acupressure [17].

In the UK, a 2012 systematic review exploring the prevalence of complementary therapies found that approximately 1 in 4 individuals taking prescription medicines also used some form of complementary therapy. This was compared with 4 in 10 adults in the USA and 1 in 2 in Australia, although the highest rates were in East Asian countries [20]. A 2018 survey in England with 4862 participants revealed that 16% had received complementary therapies and 68% of those were experiencing musculoskeletal pain (predominantly back pain) [16]. It was observed that in England, use of complementary therapies is higher in women, those in higher socioeconomic groups (i.e., those who can afford to pay) and in those living in the south of the country [16]. There is an emphasis on manual therapies (massage, osteopathy, and chiropractic), acupuncture, and yoga amongst participants. Very few episodes of complementary therapy treatments were provided free by the National Health Service (NHS).

### 3.3 Why Individuals Use Complementary Approaches to Manage Chronic Pain

The reasons why patients seek to use complementary therapies are multifaceted. One study revealed that patients use these approaches because they have a philosophical congruence with them, agreeing with the idea of holistic balance, or self‐healing, and feel dissatisfaction with conventional medicine [21]. Patients also felt that they needed to take greater control over their own health and treatment. A qualitative study on people living with of low back pain indicated that individuals sought complementary therapies with the expectations of pain relief, improvement in function, improved fitness and improved wellbeing [22]. Whilst expectation and belief might play a part in relieving pain, patients also felt that improvements in other domains (e.g., mood, quality of life) might indirectly help relieve their pain [22]. Other reasons for accessing complementary therapies include a recommendation from a General Practitioner (GP) or the presence of more than one medical condition, or an associated psychological problem, such as depression [23]. It is also likely that the availability, cost, convenience and access are also important factors [24].

In a systematic review of 231 publications, including qualitative, quantitative and mixed‐methods studies, it was found that the three main reasons for using complementary therapies were having an expectation of benefits of complementary therapies, a dissatisfaction with conventional medicine and having a perception that complementary therapies were safe [25]. However, this can vary considerably depending on nationality due to differences in religion, tradition and culture.

It has been suggested that supported self‐management programmes provide a means of helping patients with chronic pain manage their condition with support from healthcare professionals, possibly through social prescribing and group or individual therapies [26, 27]. Some complementary therapies can be self‐delivered after instruction or delivered via audio or video presentation and lend themselves to self‐management of pain (Table 1). Self‐management is thought to promote positive feelings, a sense of agency or control over the problem, and an increased confidence in self‐coping [28], along with the advantage of having little, cost to the individual. Examples of complementary therapies which could be self‐delivered include relaxation therapy, mindfulness meditation and yoga.

## 4 Approaches to Evaluating the Efficacy of Complementary Therapies for Chronic Pain

The experience of chronic pain is complex. Each patient experiencing chronic pain ascribes individual meanings to their pain and thus will have different ways of coping with it [29]. Contemporary views on pain management call for an integrated approach, as promoted by the International Association for the Study of Pain (IASP) Global Year of Pain 2023 theme on ‘integrative pain care’ (https://www.iasp-pain.org/). However, integrative approaches to chronic pain management are often grounded in biomedical mechanisms, with an emphasis on actual (or potential) tissue damage, diverting attention towards the development of interventions targeting discrete tissues [30]. These interventions do not focus on the *lived experience* of the whole person, which incorporates the sensory, emotional, cognitive, and social components of this condition [31, 32].

A more recent shift ‘upstream’, towards an understanding of the psychosocial aspects of pain draws attention to psychotherapeutic approaches and lifestyle adjustment such as meaningful activities (movement) and healthy diet, yet the burden of chronic pain remains [33].

There is a growing recognition of the need for a whole‐systems approach to population health and well‐being [30, 34]. This requires a cultural transformation which diverts focus away from specific diseases or tissue damage towards whole person health and well‐being aligned with an individual's aspirations, meaning and purpose in life. Advocates of complementary therapies claim that they provide a collaborative, whole person approach to healing [35]. Complementary therapies are unique in terms of their constituent components, their mechanisms of action (biopsychosocial) and the context in which they are delivered [36]. A whole‐person view of chronic pain acknowledges the sensory, cognitive, emotional, and social components of the pain experience [32]. A recent shift towards an understanding of the psychosocial aspects of pain has led to the delivery of other interventions such as exercise, diet and psychotherapeutic interventions all of which add to the increasing range of approaches that have still failed to reduce the burden of chronic pain [37, 38, 39, 40].

The RCT design was originally developed to evaluate pharmaceutical interventions where cause and effect were isolated, often using reductionist outcomes, such as pain intensity. Even where outcomes are ostensibly objective, at best they can only give predictions of effect [41]. The whole premise of the RCT model is to isolate cause and effect which can then be generalised across populations to inform healthcare policy. However, the complexity of the RCT process and the number of different assumptions, sources of bias and limitations introduced during the trial process can lead to an oversimplified and reductionist approach which may produce findings that are not robust or reproducible [42, 43]. This is especially true in the field of pain. For example, there are inconsistencies in the findings in RCTs investigating the efficacy of analgesic drugs either as monotherapy or in combination, resulting in inconclusive findings in systematic reviews and meta‐analyses [44]. Outcomes such as pain intensity, are subjective variables that are dynamic in nature. The problem is compounded when we add in methodological shortcomings and failure to produce evidence of unintended effects (e.g., harms or adverse events) [42].

The use of conventional RCT methodology for the evaluation of complementary therapies remains a valuable option where answers to specific causal questions is required. However, reliance on conventional efficacy‐focused RCTs are unable to capture the broader holistic outcomes associated with complementary therapies by focusing solely on the intensity or severity of pain and ignoring contextual mechanisms. This approach may undervalue broader patient experiences and should therefore be used as only one element of a complex intervention strategy. We suggest that we need to re‐focus our efforts beyond the evaluation pain and look to other outcomes which more readily reflect the milieu of responses to, and expectations of, complementary therapies. Patient responses to complementary therapies are outcomes of interest and this ‘meaning response’, as described by Segar, is important due to the neurological and physiological changes associated with it [45]. These changes have often been referred to (and frequently dismissed) as the ‘placebo response’ and include a multitude of psychosocial factors such as expectancy, conditioning, learning, reward and motivation [46]. The neurobiological effects induced by these mechanisms include activation of neural systems that release endogenous opioids, dopamine and cholecystokinin induced by expectation of analgesia. This has also been found to reduce activity in key brain regions such as the thalamus and insula [47]. More recently these neurobiological changes have been found to extend to the modulation of endocrine and immune system responses [48].

Interestingly, the expectations of therapists have also been found to be important in eliciting similar neurobiological effects [46]. It has been observed that interventions with more elaborate rituals result in similar outcomes for both real treatment and sham interventions, as does the promise of individualised treatment, suggesting that expectancy effects are powerful tools in maximising responses to any intervention [49, 50]. This goes some way to providing an explanation as to how both complementary therapies and their sham or placebo alternatives within RCTs appear to have similar neurobiological effects, which further frustrates the quest for evidence of efficacy [50].

To ensure that the broader effects of complementary therapies are captured, we therefore suggest that research designs other than the traditional RCT might be utilised; these might include pragmatic trials and mixed methods designs, or the use of process evaluations alongside programme theory approaches.

## 5 Establishing the Utility and Benefits of Complementary Therapies for Chronic Pain

To explain how each unique element of complementary therapies interact and produce outcomes we need to identify and understand them as holistic mediators of change. If we consider complementary therapies as akin to complex interventions due to their multifaceted nature and unique individual experiences and contexts, this provides a starting point for the development of appropriate methods of evaluation. This enables us to refocus our approach away from efficacy and more towards evaluating the meaning, responses to, and utility of complementary therapies, enabling us to highlight outcomes which reflect these responses. In other words, this approach focuses less on whether an intervention *works* and more on its *impact* [51].

The Medical Research Council (MRC) has produced a framework for the development and evaluation of complex interventions [51]. The recommendation is to use programme theory to explain how and why an intervention is expected to work. This process requires us to determine the key components (or core elements) of the intervention, how they interact, what mechanisms are thought to be acting and the contextual features influencing and influenced by these mechanisms [52]. The development of programme theory is evidence‐based and can continually be refined with the input of users of the intervention and other stakeholders, such as therapists [51]. The refined programme theory can then be applied to the diverse contexts of everyday life and is often visually illustrated within a logic model. A logic model shows how interventions can be evaluated within a variety of ‘real‐life’ situations, cultures and traditions helping us to gain an understanding of the potential mechanisms of action leading to the *impact* of each intervention. Figure 1 shows a simple illustration of how this can be achieved and how appropriate studies can then be designed which incorporate ways of evaluating the outputs which reflect the lived experience of individuals who have received complementary therapies.

**Figure 1:**
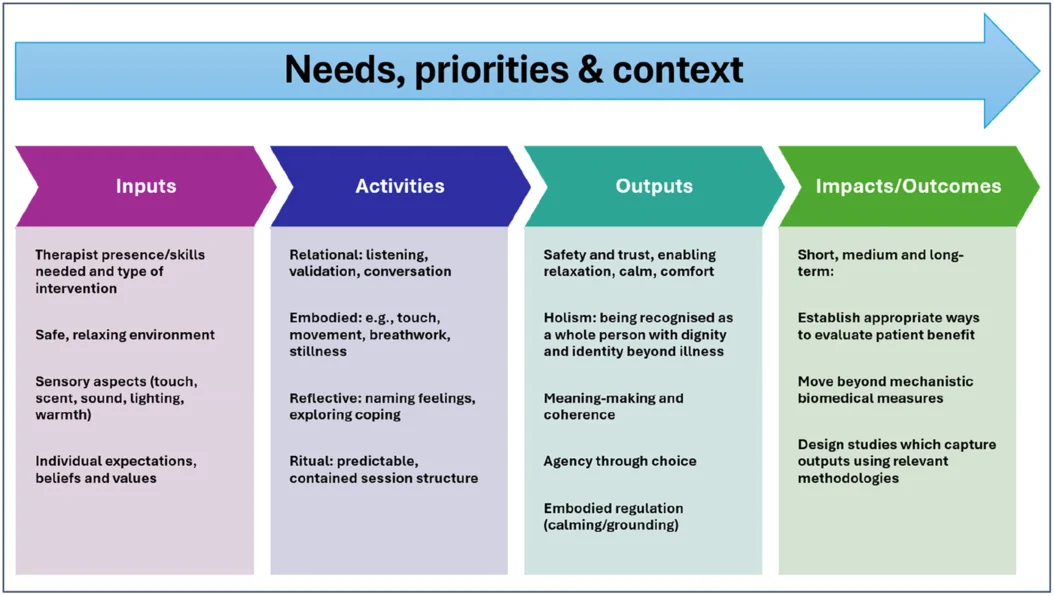
Example of a simple logic model.

### 5.1 Potential Outcome Domains Emerging From Programme Theory

Outcomes should emerge from a programme theory development and stakeholder engagement rather than being predetermined and definitive. Examples of possible outcome domains within complementary therapies include comprehensibility, manageability, meaningfulness of the treatment, lived experience, and self‐efficacy (Figure 1). These domains are presented as examples of outcomes which might emerge from programme development, rather than being pre‐stipulated.

Using this approach towards establishing the components of complementary therapies, we can identify a range of potential impacts on the recipient which will provide essential elements of the evaluation process [53]. The identification of the active components and unique identities for each complementary therapy will facilitate the development of a programme theory which then feeds into a logic model. These unique components might be physiological, environmental, social (i.e., the patient/therapist interface) or philosophical (underlying belief‐systems). The logic model will describe how impacts or outcomes are achieved, and which elements of the intervention contribute to this, thus enabling us to identify or develop suitable outcome measures. As a first step in this process, we have separated the complementary therapies listed in Table 1 into their unique components which are listed Table 2. The unique identities and components for each of the complementary therapies described in Table 2 could be refined further by exploring the meanings and perceived benefits ascribed to these approaches by individuals living with chronic pain.

**Table 2:** A summary of unique characteristics of selected complementary therapies.

Therapy	Physiological	Contextual	Relational	Philosophical	Selected references
**Massage**	Touch, pressure, sensory stimulation	Quiet environment	Therapeutic touch	Holistic wellbeing	Mak et al. [54] Posadzki, et al. [55]
**Reflexology**	Touch stimulation according to body mapping	Relaxing environment	Therapeutic touch Therapist interaction	Has roots in TCM. Involves reflex maps and energy balance in the body	Ernst et al. [56] Stephenson & Dalton [57]
**Acupuncture**	Needle stimulation	Ritual and treatment setting	Therapist interaction	Qi and meridian theory (TCM) or western medical approaches (WMA)	Paley & Johnson [58] Vickers et al. [59]
**Aromatherapy**	Olfactory stimulation via essential oils.	Relaxing environment	Therapist interaction or self‐delivery	Emphasis on balance between physical health, spiritual needs and wellbeing	Lee et al. [60] Scuteri et al. [61]
**Mindfulness/meditation**	Attention regulation	Quiet and relaxing environment	Therapist interaction or self‐delivery	Contemplative traditions, including Buddhism	Ball et al. [62] Hilton et al. [63]
**Yoga, Tai Chi or Qi Gong**	Bodily movement and stretching tissues	Relaxing environment	Teacher facilitation or self‐delivery	Holistic TCM movement practices integrating posture, breath and mental focus	Zhu et al. [64] Martinez‐Calderon et al. [65]
**Creative arts therapies**	Various depending on activity; e.g., agility, coordination, fine motor skills.	Individual or group setting.	Teacher facilitation or self‐delivery	Creative process as a primary vehicle for healing, meaning making and self‐discovery	Harasymchuk et al. [66] Raudenská et al. [67] Matthew Lee et al. [68]

Abbreviations: TCM, Traditional Chinese Medicine; WMA, Western Medical Approaches

The unique components proposed for complementary therapies vary considerably in their evidential basis. Some are supported by experimental and neurophysiological research, whereas others remain theoretical hypotheses, traditional explanatory models, or practitioner narratives. The purpose of Table 2 is not to validate particular mechanisms but to identify components that may contribute to patient experiences and outcomes, and which may subsequently inform programme theory development.

We suggest that the next stage in this process will be to work closely with individuals living with chronic pain, to refine and add to the unique elements identified in Table 2 and create a list of unique meaning‐responses for each intervention. We propose that the term ‘meaning‐response’ should not be confused with placebo effects because traditionally, the term ‘placebo’ means ‘inert’ and therefore should not be used to label anything that could be described as a therapeutic effect [46, 69]. However, Moerman and Jonas argue that the concept of placebo is now used more often to describe responses which reflect the embedded symbolism and meaning within health interventions [69, 70]. The resulting ‘meaning response’ reflects the way individuals describe their experiences when exposed to those interventions. Desirable effects can still be derived from interventions which were previously thought to be inert (placebos), such as sham treatments, and all are driven by meaning. To help resolve this paradox, we should consider physiological sequalae occurring because of the meaning responses to so‐called placebos, such as the release of endorphins or the hormonal responses to treatment, which have been proposed as being the ability of the body to heal itself [45, 70, 71]. Studies using naloxone, an antagonist to endogenous opioids, have shown some reversal of these analgesic effects [72]. The role of context, patient expectancy/belief and classical conditioning describe two of the mechanisms thought to underlie meaning responses and have been substantiated using functional Magnetic Resonance Imaging (MRI) scans [73]. It needs to be recognised that factors previously excluded and controlled within RCTs, such as the therapist/patient relationship, environmental or contextual factors, non‐specific effects of care and personal beliefs and expectations should be embraced as part of a therapeutic process which takes place over time [45, 74]. We need to consider what constitutes a clinically meaningful overall benefit and how this should best be evaluated.

### 5.2 Safety, Burden and Ethics

Any comprehensive evaluation framework must capture harms and burdens alongside benefits, and these should be specified within the programme theory and logic model. Intervention‐specific adverse events for each complementary therapy should be distinguished from wider clinical concerns.

Invasive interventions such as acupuncture introduce potential for adverse events including bleeding, bruising or infection, and the potential for these occurrences should be explained to patients prior to administration. In the case of acupuncture, the occurrence of serious adverse events is rare, but have been recorded in a small fraction of cases [75]. These adverse events are almost always preventable with adequate practitioner knowledge, training and clinical guidelines.

There is potential for bruising and discomfort during other manual therapies such as massage and reflexology. During movement‐based therapies such as yoga, tai chi and some of the creative arts may result in temporary exacerbation of symptoms, falls or injury. Their likelihood and severity of adverse events will vary according to the therapy, mode of delivery, practitioner competence and the individual's clinical circumstances. When evaluating these interventions, practitioners should record the nature, timing, severity, duration and management of adverse events, rather than simply assuming that the intervention is safe.

Burden extends beyond adverse events. To achieve their intended goals, complementary therapies often require repeated appointments, travel, sustained home practice and substantial financial expenditure [76]. Provision of many complementary therapies is not available through the NHS, and where it is, this provision is usually limited to a prescribed number of sessions [77]. These burdens are especially significant for individuals with limited mobility, energy, access to transport and finite access to funds. Attrition, non‐adherence and treatment discontinuation may therefore be an indicator of excessive burden rather than a lack of motivation and this should be explored alongside patient‐reported acceptability.

Ethical concerns include misleading practitioner claims, failure to recognise contraindications, poor communication with conventional healthcare providers, or use of complementary therapies in place of indicated medical care may delay diagnosis or effective treatment [78, 79]. These risks may be reduced or mitigated using appropriate screening, informed consent, clear practitioner boundaries and referral pathways.

In summary, any evaluation of complementary therapies should therefore assess patient safety and burden and determine whether patient‐valued benefits are sufficient to justify the costs and risks involved [80].

## 6 Conclusions and Directions for Future Research

Complementary approaches for chronic pain continue to gain in popularity and (often anecdotally) they appear to be beneficial. However, evaluation of these benefits using RCTs to establish efficacy have not, to date, provided consistent evidence of efficacy. This method of evaluation does not reflect the way patients view and experience complementary therapies, nor does it consider other factors such as context and environment. We suggest an approach to the evaluation of complementary therapies through an exploration of the unique features of each intervention, determining what patients find important and beneficial. It is not sufficient to dismiss observed benefits solely as placebo effects, particularly when such responses involve meaningful psychological, behavioural and neurobiological changes that may contribute to patient‐valued outcomes. We need to embrace any responses to complementary therapies and reconsider their importance in managing chronic pain.

## Author Contributions

All authors conceived the study. Carole A. Paley conducted searches of the literature. Carole A. Paley and Mark I. Johnson were involved in study design. All authors analysed and interpreted the findings. Carole A. Paley and Mark I. Johnson wrote the first draft of the manuscript. All authors reviewed and edited the manuscript and approved the final version of the manuscript.

## Funding

The authors have nothing to report.

## Ethics Statement

The authors have nothing to report.

## Conflicts of Interest

The authors declare no conflicts of interest.

## Data Availability

All data generated or analysed during this study are available from the corresponding author on reasonable request.
